# Universal health insurance, health inequality and oral cancer in Taiwan

**DOI:** 10.1371/journal.pone.0205731

**Published:** 2018-10-18

**Authors:** Fuhmei Wang, Jung-Der Wang, Yu-Wen Hung

**Affiliations:** 1 Department of Economics, National Cheng Kung University, Tainan, Taiwan; 2 National Cheng Kung University, Department of Public Health, College of Medicine, Tainan, Taiwan; Cancer Research Malaysia, MALAYSIA

## Abstract

**Introduction:**

The introduction of universal health insurance coverage aims to provide equal accessibility and affordability of health care, but whether such a policy eliminates health inequalities has not been conclusively determined. This research aims to examine the healthcare outcomes of oral cancer and determine whether the universal coverage system in Taiwan has reduced health inequality.

**Methods:**

Linking the databases of the National Cancer Registry with the National Mortality Registry in Taiwan, we stratified patients with oral squamous cell carcinoma by gender and income to estimate the incidence rate, cumulative incidence rate aged from 20 to 79 (CIR20-79), life expectancy, and expected years of life lost (EYLL). The difficulties with asymmetries and short follow-up periods were resolved through applying survival analysis extrapolation methods.

**Results:**

While all people showed a general improvement in life expectancy after the introduction of the NHI, the estimated change in EYLL’s of the high-, middle-, and low-income female patients were found to have +0.3, -0.5 and -7 years of EYLL, respectively, indicating a reduction in health inequality. Improvements for the male patients were unremarkable. There was no drop in the CIR20-79 of oral cancer in disadvantaged groups as in those with higher incomes.

**Conclusions:**

Universal coverage alone may not reduce health inequality across different income groups for oral cancer unless effective preventive measures are implemented for economically disadvantaged regions.

## Introduction

Regardless of measurements of economic prosperity and health outcomes, health inequalities still exist among different geographic and economic groups in both developed and developing economies [1, 2]. Healthcare provisions should thus be designed to improve the health of a population and their living conditions, irrespective of geographical location or economic resources. A lack of health insurance is frequently viewed as the main reason for increased morbidity and mortality in populations, and is also blamed for widening gaps in health inequality. The introduction of universal health insurance coverage aims to provide equal accessibility and affordability of health care, but whether such a policy eliminates health inequalities has not been conclusively determined. Some studies have shown that a universal coverage system could reduce economic health disparities [3, 4], while others have indicated that these reductions are only of limited extent or even resulted in the opposite effect after the implementation of an NHI (National Health Insurance) system [5–8]. However, most of these studies have limited their definitions of the related health measures to mortality or life expectancy.

Public-health efforts, such as sanitation and vaccination, medical care, and economic growth improved the life expectancy in Taiwan over the period of 1990–2007 for not only oral cancer patients but also the healthy/general population. The estimation of life expectancy for patients in the literature was generally limited due to insufficient follow-up periods. However, there are methods that apply the extrapolation of survival curves in order to estimate the life expectancy and expected years of life lost (EYLL) in patients with major cancers, which usually follow a constant excess hazard after the initial period of treatment and 5–10 years of follow-up [9–11]. The survival curve of a cohort with a specific cancer can thus be estimated through the Kaplan-Meier method up to the end of follow-up, and the life expectancy can then be extrapolated using a semi-parametric method with good accuracy [12]. This estimation method has been designed to solve the difficulties related to the high-censored rates seen with most chronic diseases [13].

Taiwan introduced its universal NHI system in1995, and the implementation has become an important learning model for any country that plans to revamp or redistribute its healthcare services and medical insurance system. Despite the universal coverage that is offered, rural residents tend to have lower rates of NHI utilization than those living in cities, and thus health disparities may still exist between these groups [6].

Previous works have revealed that while Taiwan’s NHI has increased the utilization of health services, it has only reduced health disparities related to life expectancy to a limited extent [6, 8, 14]. This study thus aims to measure health inequality with regard to outcomes, for which we abstracted data collected in the cancer registry for patients diagnosed with oral squamous cell carcinoma. We chose this disease because the World Health Organization (WHO) has stated that cancer has now reached epidemic proportions, with more than 70% of all cancer deaths occurring in low and middle income groups. Based on statistics for cancer incidence rates collected by the Taiwan Cancer Registry over the last decade, the leading five cancers for males in Taiwan are, in order, liver cancer, lung cancer, colon-rectal cancer, oral cancer, and stomach cancer [15]. Income data can be used to capture the health disparities in cancer epidemiology [16], and oral squamous cell carcinoma is primarily associated with chewing betel quid among males of the lower income population, a disease that has become more widespread throughout Taiwan [17–19].

By analyzing national datasets and linking them together, this study aims to examine whether the universal coverage provided by Taiwan’s NHI actually reduces health inequities with regard to oral cancer. In brief, we offer new evidence in the following aspects: First, our outcome measures include both life expectancy and EYLL, and thus permit us to examine the health benefits after accounting for age at diagnosis and/or lead time bias. Second, this study is among the first to examine how differences in gender as well as income, classified by residential area, impact the treatment effects related to universal health insurance. Moreover, the use of area of residence to represent socio-economic status has been recognized as a valid way to study inequalities in cancer incidence [16]. Third, we determined the lifelong risk, *i*.*e*., from age 20 to 79, of oral squamous cell carcinoma to pave the way for future cost-effectiveness analyses intended to prevent and improve health disparities in this context.

## Methods

### Primary data

The primary data were collected from the Taiwan Cancer Registry. The diagnostic criteria for oral squamous cell carcinoma were based on the Classification of Diseases for Oncology, Field Trial Edition [20].

### Time period

Although the Taiwan Cancer Registry started in 1979, the data became more complete after 1990. The exclusion of data from 1995-1997was to avoid potential confounding factors due to the lack of comprehensiveness in data collection when the NHI system was first implemented in April of 1995. The study periods included the periods of 1990–1994, representing the phase before the introduction of the NHI, and 1998–2007, representing the phase after its implementation. Although the observed periods are asymmetric, the difficulties with asymmetries and short follow-up periods were resolved through applying survival analysis extrapolation methods, as mentioned above. Because the Taiwan Cancer Registry records patients according to the places in which they reside, this research ranks the population according to household disposable income and classifies them into high, middle, and low income classes. Areas of residence were ranked according to the Survey of Family Income and Expenditure in Taiwan [21].

### Extrapolation of long-term survival and estimation of life expectancy

Previous research has documented the validity of this semi-parametric extrapolation of long-term survival and estimation of life expectancy for cancers of 17 different organ-systems, including oral cancer [12]. A recent review conducted by the Medical Research Council of the U.K. on fitting different extrapolation models for survival research also approved this method [13]. In brief, we classified the survival status of all cases through cross-linkage with the National Mortality Registry in Taiwan to the end of the follow-up periods, December 31, 1994, and December 31, 2009, representing the periods before and after the introduction of the NHI. The Kaplan-Meier method was used to estimate the survival curve based on follow-up data from the periods 1990–1994 and 1998–2007. Simultaneously, we applied the Monte Carlo method to the national vital statistics to generate the lifetime survival curve of the age- and gender-matched reference population. Incorporating the lifetime survival curve of the age-, sex, and calendar year-matched referents, we extrapolated the survival curve of the index population to lifetime under the assumption of constant excess hazard [10], which generally holds for diseases causing premature mortality, especially among cancer cohorts [12]. Then, we summed up the total area under the lifetime survival curve to obtain the life expectancy.

### Expected years of life lost

The difference between the life expectancies of patients and their age-, sex, and calendar year-matched referents was the expected years of life lost (EYLL) related to oral squamous cell carcinoma. To extrapolate the long-term survival curve and calculate the life expectancy of the focal cohort, we conducted not only a logit transformation for the survival ratios of both the cancer cohort and reference population, but also a linear regression to estimate the slope for extrapolation [9, 10]. This research further used a bootstrap method to conduct 100 repeated random samplings of the cancer cohort to estimate the mean and standard error by using the iSQoL software program (integrating survival with quality of life), which can be freely downloaded (www.stat.sinica.edu.tw/jshwang).

### Estimation of cumulated incidence rate_20-79_ (CIR_20-79_)

The 10-year average incidence rate was defined as the number of patients with oral squamous cell carcinoma divided by the population with the same age, gender, and income in the year of diagnosis. Accumulating the 10-year incidence rates (IR) over the ages of 20–79 years, we obtained the CIR_20-79_ through the following formula:

$$CIR_{0-t}=1-e^{-\sum_{i}(IR_{i})(\Delta t_{i})}$$

where IRi is the age-specific incidence rate for the stratum *i*, Δt_i_ = 10. This represents a person’s lifetime (from age 20 to 79) probability of developing oral squamous cell carcinoma, if s/he has not died of other diseases. Thus, it can be interpreted as a lifetime risk.

### Ethics statements

The funders had no role in the study design, data collection and analysis, decision to publish, or preparation of the manuscript with the registered number of 201006061R approved by the Research Ethics Committee of National Taiwan University Hospital, and this study was fully ratified in the 6^th^ meeting of the Research Ethics Committee of National Taiwan University Hospital on July 2, 2010. On 6^th^ July, 2017, our named ethics committee specifically approved this study.

## Results

### Accessibility of health care results in limited improvements in health inequality

Table 1 summarizes the demographic characteristics of patients with oral squamous cell carcinoma. Although the average ages at diagnosis appear similar among males in different income groups, the female patients in the low-income group were generally diagnosed at older ages, both before and after the introduction of the NHI. The censored rates of follow-up were lower in low income areas both before and after the establishment of the NHI. Those of male patients, however, were similar among different income groups before and after NHI implementation. Before the implementation of the NHI, the diagnosed cases for male patients in high, middle, and low income groups in order were 175, 146, and 208; for female patients in high, middle, and low income groups in order were 1496, 1341, and 2370. After the implementation of the NHI, the diagnosed cases for male patients in high, middle, and low income groups in order were 834, 827, and 1172; for female patients in high, middle, and low income groups in order were 9023, 9959, and 15526. The implementation of the NHI increased the diagnosed cases among different income groups because universal coverage increased the affordability of health care.

**Table 1:** Demographic characteristics of patients with oral squamous cell carcinoma for high, middle, and low income groups stratified by gender, before versus after NHI (National Health Insurance) implementation.

Stratification		Before NHI 5-year follow-up			After NHI 12-year follow up		
	Classes	Cases	Age diagnosed (SD)*	Censored rate (%)	Cases	Age diagnosed (SD)*	Censored rate (%)
Female	High	175	57.2 (13.9)	68.4	834	58.4 (15.0)	64.4
	Middle	146	56.8 (13.9)	65.1	827	57.6 (15.0)	62.3
	Low	208	59.4 (13.6)	59.4	1172	60.5 (14.4)	56.7
Male	High	1496	53.1 (12.5)	58.3	9023	54.4 (12.1)	53.6
	Middle	1341	52.7 (12.4)	58.9	9959	51.9 (11.7)	54.1
	Low	2370	53.4 (11.9)	57.6	15526	52.7 (11.7)	52

*SD: Standard Deviation.

### Validity of estimation results

**Fig 1:**
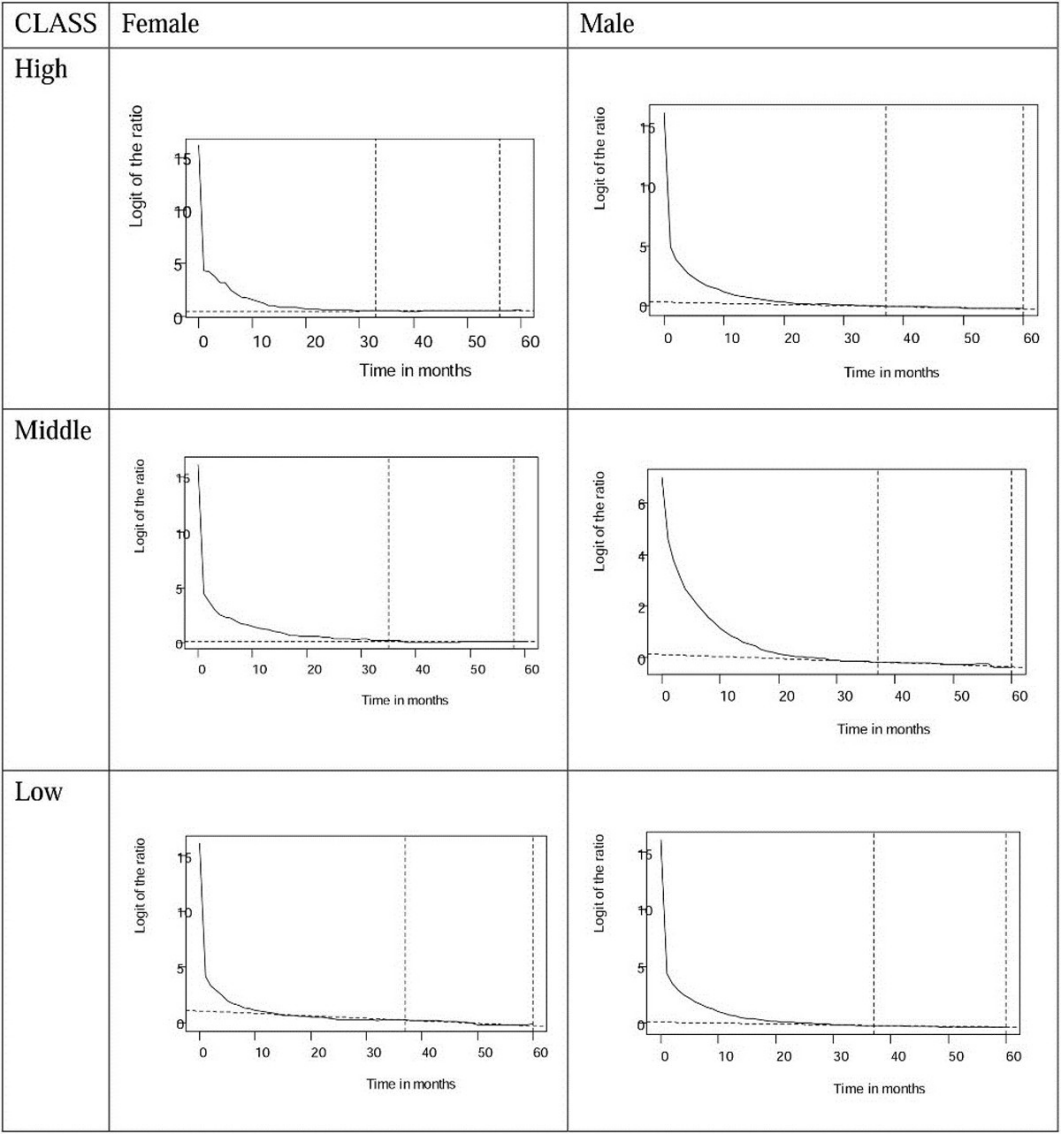
Logit transformation of survival ratios between oral cancer patients and age-gender-matched referents from the life table of Taiwan showing the convergence to a straight line and stable slope (the segment near the end indicated by dotted lines), during 5 years follow-up before the establishment of NHI (National Health Insurance) in 1995 by high, middle, and low income ranked.

**Fig 2:**
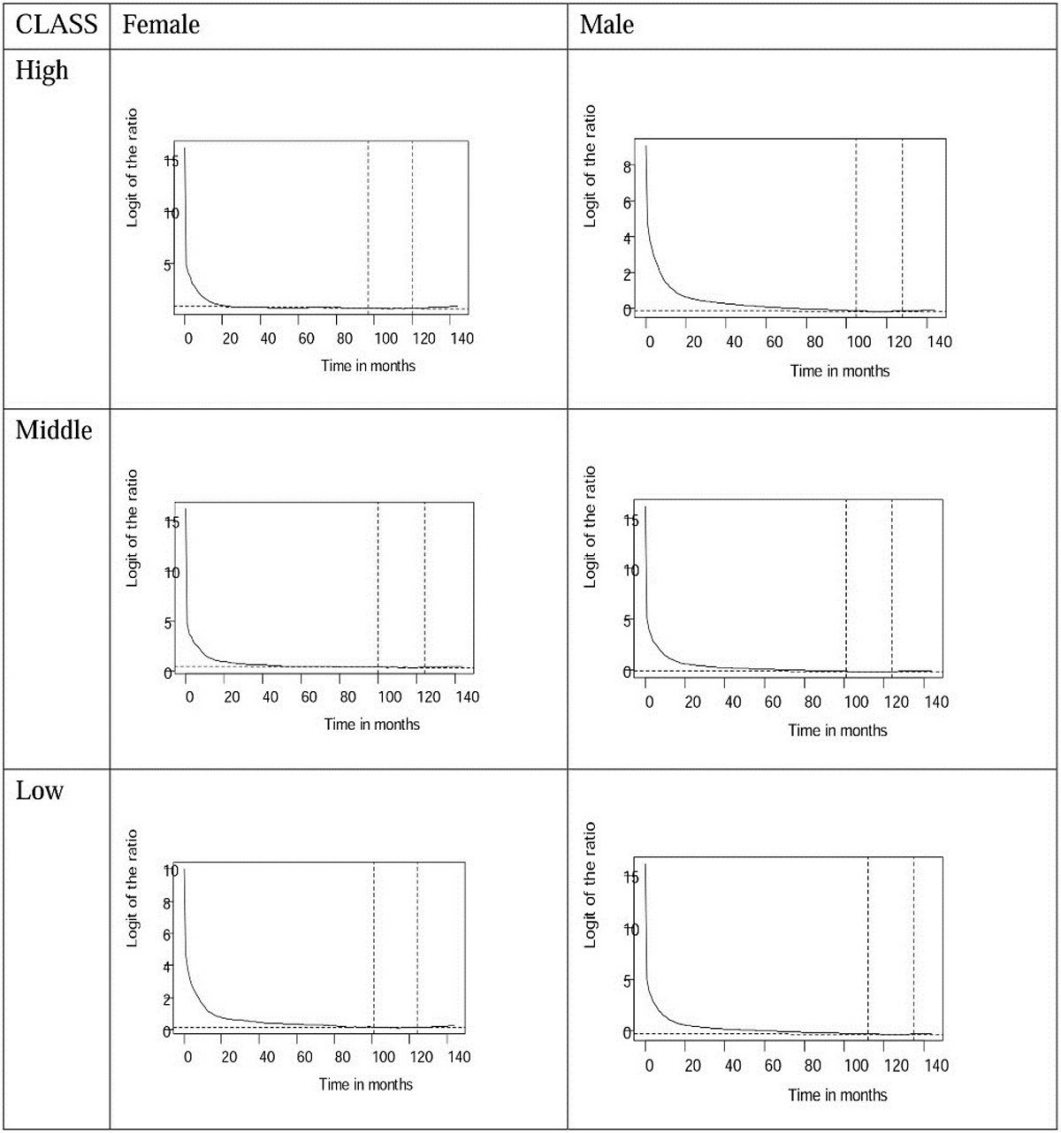
Logit transformation of survival ratios between oral cancer patients and age-gender-matched referents from the life table of Taiwan showing the convergence to a straight line and stable slope (the segment near the end indicated by dotted lines), during 12 years follow-up before the establishment of NHI (National Health Insurance) in 1995 by high, middle, and low income ranked.

Through logit transformation of the survival ratios of patients with oral cancer and age- and gender-matched referents, we found that all of the transformation converge to stable slopes, as shown in Figs 1 and 2. The trends on the diagnostic plots reveal that all cohorts of patients with oral squamous cell carcinoma in different classes follow a constant excess hazard at the end of five and 12 years of follow-up, before and after the implementation of the NHI. The estimation results are thus valid.

### EYLLs accurately assess health inequality

**Fig 3:**
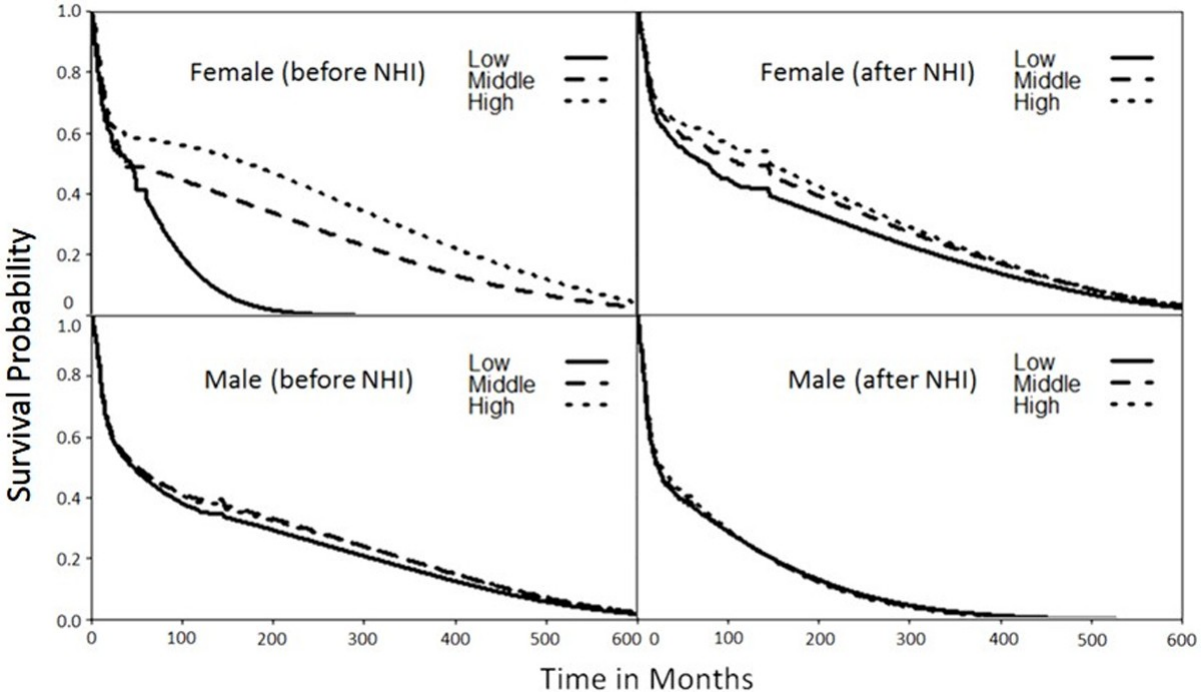
Extrapolated survival curves of patients with oral squamous cell carcinoma diagnosed for all three groups stratified by gender and before versus after NHI implementation (H:High, M:Middle, and L:Low income ranked group).

Fig 3 shows the estimated survival curves for all three groups stratified by gender, before versus after the implementation of the NHI. Table 2 shows that the life expectancies of all patients with oral cancer improved after the introduction of the NHI. Before implementation of the NHI, female patients in the high-, middle-, and low-income groups had life expectancies of 14.9, 13, and 4.6 years, respectively, which corresponded with 8.4, 10.7, and 17.0 years of EYLL after adjustment for age at diagnosis. After the NHI was introduced, the estimated EYLL’s became 8.7, 10.2, and 10.0 years for the high, middle, and low-income groups. Although the high-income females still had the advantage of the smallest loss of life expectancy, the low-income group showed a 7.0 (= 17.0–10.0) year reduction in health inequality. Table 2 further compares the EYLLs of the middle and low income oral cancer patients to those of high income patients before and after NHI through Z tests. Before the implementation of NHI, the EYLLs of low income female patients were significantly greater than those of the high income female patients by 8.6 years. The standard error of mean (SEM) for EYLL was computed through boot-trap method. Since the life expectancy of the simulated age-, sex-, and calendar-year matched referents was based on national life tables, its SEM was generally very small. Thus, the SEM of EYLL was mainly decided by the SEM (and sample size) of the index cohort, as summarized in the Table 2. Therefore, the test is valid and reproducible.

**Table 2:** Comparison of life expectancy (LE, in years) and expected years of life loss (EYLL) for patients with oral squamous cell carcinoma for high, middle, and low income groups stratified by gender, before versus after NHI implementation.

5-year follow-up		Before NHI		Comparison with high classes	Referents	After NHI		Comparison with high classes	Referents	Changes in EYLL
	Classes	LE (SE)	EYLL		LE(SE)	LE (SE)*	EYLL		LE(SE)	
Female	High	14.9 (3.6)	8.4	0	23.4 (0.0)	16.3 (2.0)	8.7	0	25.0 (0.0)	+0.3
	Middle	13.0 (3.3)	10.7	2.3	23.7 (0.0)	15.4 (1.9)	10.2	1.5	25.6 (0.0)	-0.5
	Low	4.60 (2.2)	17.0	8.6*	21.6 (0.0)	13.3 (1.6)	10.0	1.3	23.4 (0.0)	-7
Male	High	6.5 (1.3)	17.2	0	23.7 (0.0)	13.0 (0.5)	13.4	0	26.4 (0.0)	-3.8
	Middle	6.5 (1.9)	17.6	0.4	24.0 (0.0)	13.1 (0.6)	13.7	1.3	26.8 (0.0)	-3.9
	Low	6.5 (1.2)	16.9	0.3	23.4 (0.0)	11.8 (0.5)	14.3	0.9	26.1 (0.0)	-2.6

Note: SE is standard error;

*: p < 0.05

Regardless of income rank, male patients with oral cancer had almost the same life expectancies of 6.5 and 12–13 years, respectively, before and after the introduction of the NHI, which corresponded to 13 and 14 years of EYLL before and after the NHI (Table 2 and Fig 3). This indicated no or less disparity among male patients for the different levels of income. However, introducing the NHI improved the life expectancies of all male patients by about 5.3–6.5 years, which corresponded to a reduction in EYLL of about 2.6–3.9 years. Moreover, male patients in the low-ranked income group had the highest EYLL among all male patients after the NHI was introduced.

**Fig 4:**
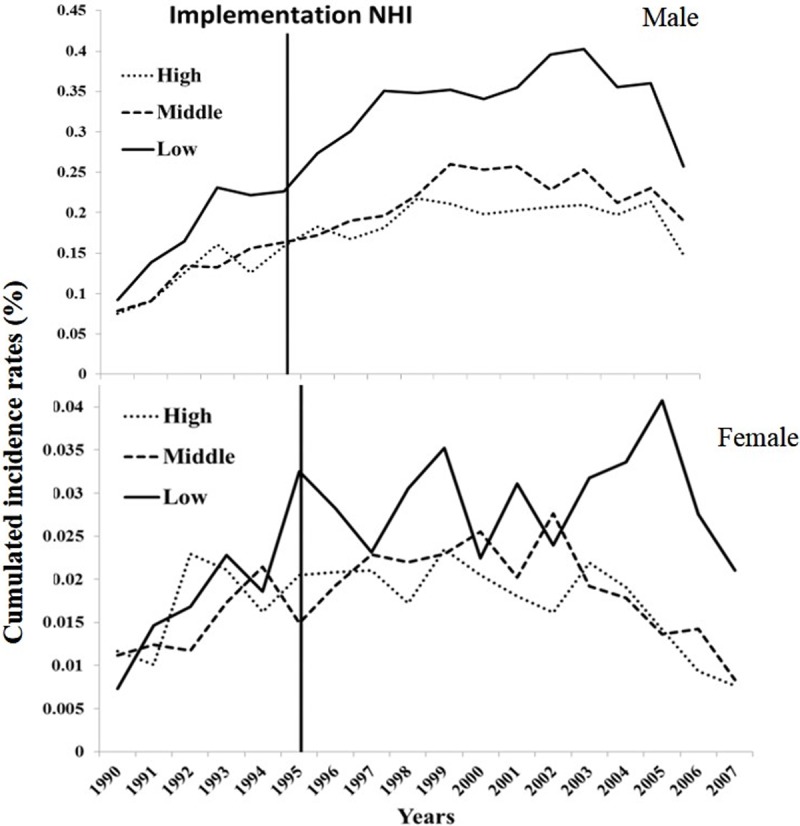
Lifetime cumulated incidence rates, aged from 20 to 79 (CIR_20-79_), of oral squamous cell carcinoma for males (upper panel) and females (lower panel) stratified by income ranked groups.

Based on the trends of cumulated incidence rates (CIR_20-79_), Fig 4 shows that the low-income groups in Taiwan suffered a persistent high risk of oral squamous cell carcinoma, and the gap between the income groups increased after 2003–4. The low-income groups, and especially the male population, were more likely than the high- and middle-income populations to have a higher incidence of oral cancer, as has been previously reported [17, 19].

### Only low-income females show a tremendous improvement in EYLL

Life expectancy showed general improvement after the introduction of the NHI. This was not only seen in oral cancer patients but also to the healthy/general population during the study periods. Based on the data of vital statistics from the Ministry of Interior in Taiwan, improvements in health care through the introduction of a NHI system, was reflected in the life expectancy of the general population at birth, which increased from 74 years in 1990 (before NHI) to 78.38 years in 2007 (after NHI). To control the different age, sex, and calendar year of occurrence of oral cancer, we simulated age-, sex-, and calendar year-matched referents for every new case of oral cancer from the vital statistics of Taiwan. Namely, we had a control group composed of age-, sex-, and calendar year-matched referents simulated from the life tables of the general population of Taiwan. And the expected years of life lost (EYLLs) was calculated from the difference between the oral cancer cohort and the age-, sex-, and calendar year-matched referents, which actively adjusted for the different life expectancy of each case according to his/her age, sex, and calendar year of diagnosis.

**Fig 5:**
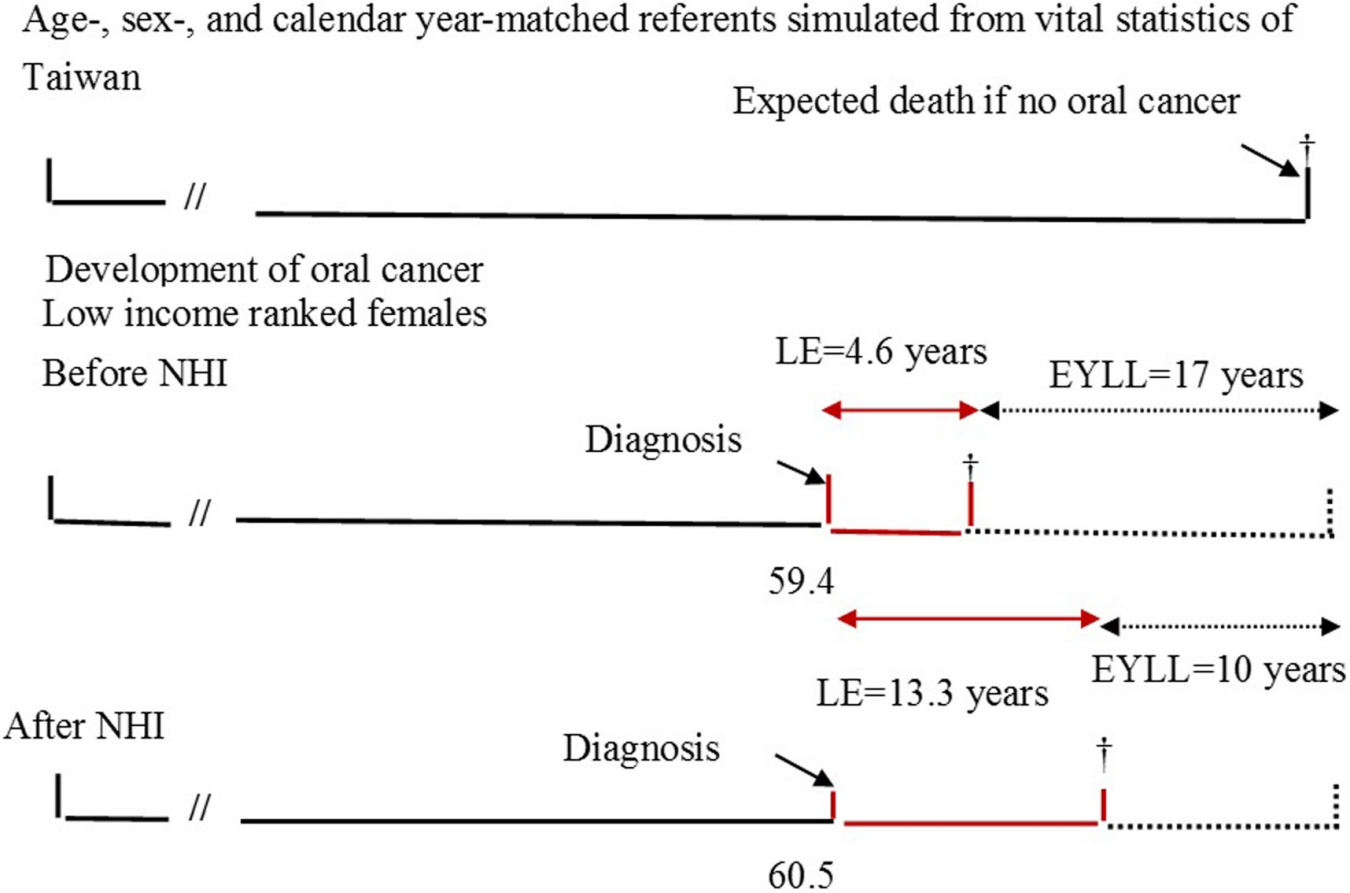
Before NHI: A case of oral squamous cell carcinoma (OSCC) for low income ranked female is on average diagnosed at age of 59.4 (Table 1), while that after National Health Insurance (NHI) is at age of 60.5. The gain in life expectancy (LE) between before and after NHI would be 13.3–4.6 = 8.7 years. However, if we take two different ages and calendar periods of diagnosis into account by comparing the difference in expected years of life lost (EYLLs) of the above two systems, the potential gain would be 17–10 = 7 years. † indicates mortality.

To clarify the concept of EYLL, Fig 5 shows the potential gains of low income females by comparing the difference in EYLLs for oral cancers diagnosed before NHI versus after NHI. Because the EYLL was adjusted for different ages and calendar years at diagnosis, the difference in EYLLs would also be adjusted for such confounders. According to Table 2, the difference in EYLLs before and after NHI would be 17–10 = 7 years.

## Discussion

### Link between accessibility and health improvement

Table 2 shows that the implementation of the NHI in Taiwan has been accompanied by improved life expectancy for all patients with oral cancer. Surprisingly, the EYLL of low-income female patients improved the most, whereas those of male patients decreased similarly among all income groups. Moreover, the dynamic changes in CIR_20-79_ shown in Fig 4 reveal a persistently high oral cancer risk among low income males and females, while both middle and high income groups faced less risk after 2003, when the Health Promotion Administration launched a national smoking cessation program [22]. These findings can be supported by the following arguments: First, we applied a novel method to extrapolate the lifetime survival curve, the accuracy of which has been validated by demonstrating the existence of a constant excess hazard (Figs 1 and 2). Second, instead of directly comparing life expectancies for three income groups, we estimated the EYLL by subtracting the life expectancy of the cancer cohort from the age- and sex-matched referents generated from the hazard functions of the national life tables. Through such a difference-in-differences design, our comparison was adjusted for different age distributions and/or possible lead time biases among the three groups [23, 24]. Third, we calculated the CIR_20-79_ for comparison of lifetime risks among the three groups. Since this indicator inherently adjusts for different age distributions [25], the direct comparison of trends over time is also valid. We thus tentatively conclude that the health effects from universal coverage do not spread equally across different income classes, and such a system may not necessarily reduce the occurrence rate of oral cancer if no deliberate inclusion and promotion of effective prevention programs are simultaneously implemented.

Our findings suggest that the NHI in Taiwan has positively affected healthcare utilization, although with only limited or partial success with regard to reducing health inequalities. The reduction in health inequality is mainly noticeable among low-income female patients, although it should be noted that increasing healthcare accessibility did improve the life expectancy for all patients with oral squamous cell carcinoma. After implementation of the NHI, the numbers of both male and female cancer patients apparently increased (Table 1), since all citizens are now entitled to healthcare with no co-payment for services directly related to cancer. Before NHI, patients might have been hesitant to accept a cancer diagnoses, due to the cost of treatment, and thus might not have been included in the Taiwan Cancer Registry. After NHI, more people accepted diagnosis and treatment due to the cut in financial burden, which contributed to the increased number of oral cancer patients. However, an increased number of diagnosed cases cannot be ruled out because there was no obvious reduction in people’s habits of betel quid chewing throughout the years of observation.

As Tables 1 and 2 show, low-income male patients presented a higher increase in diagnosed cases of oral cancer, compared to middle- and high-income male patients, but a lower reduction in EYLL. This suggests that increased accessibility to healthcare services may not lead to optimal outcomes for all health equality measures in NHI programs. Indeed, using a sample of adults with cancers in the U.K. during the period of 1996–2006, one study found that survival improved for most cancers after the implementation of the Cancer Plan, but inequalities with regard to survival were still prevalent for many other cancers [26]. Accordingly, using two samples of elderly people from two countries, Germany and the United States, with a very low and significant proportion of the populations not covered by health insurance, health inequalities were observed among the elderly in both nations [7]. Therefore, access to health care might not be the only crucial factor by which to explain such inequalities.

### Incidence rates are associated with poverty

Although this study found that the life expectancy of patients improved after the implementation of the NHI, those residing in low income areas continued to experience the greatest burden or lifetime risk for oral squamous cell carcinoma, as Fig 4 shows, and this is especially true for males patients. The health burden or impact is not only associated with the consequence of the disease, as presented by EYLL, but also with the likelihood of developing oral cancer, as presented by the lifetime cumulative incidence rate. While the gaps in EYLL between low- and middle- or high-income patients became smaller, possibly because of the universal coverage offered by the NHI (Table 2), those of lifetime risks persisted or even widened (Fig 2). Although complex and interrelated factors contribute to the observed disparities in the incidence of oral squamous cell carcinoma, the most noticeable factor is associated with low economic status. Because individuals from low income areas have a much higher prevalence of betel quid chewing [17–19], we expect oral cancer may continue to rise in these counties and townships unless appropriate preventive actions and healthcare services can be effectively applied to address this population.

Monitoring incidence trends can help evaluate progress in cancer control and reinforce prevention activities. Fig 2 shows that males had a higher prevalence of oral cancer in all incomes groups when compared to females. The high proportion of cancer among males might be due to higher prevalence of alcohol consumption, cigarette smoking, and betel quid chewing [18]. The trends of incidence rates for all oral cancer cases increased over the early 1990’s. In contrast, those of the high-income group fell after 1999–2000, while those of both genders increased for low-income populations. The variations in the incidence rates among male and female populations were possibly due to the differences in cultural factors related to betel quid chewing. Annual decreases in the incidence rates among all males and females in 2007 demonstrate the most notable change in this trend. The Department of Health in Taiwan began to promote early detection for all cancers in 2003, as well as encourage smoking cessation [22], which possibly led to the decreases in incidence rates of oral cancer seen in Fig 4. However, the dynamic changes of CIR_20-79_ in females from the low economic and demographic group do not show the same downward trend as those of the high and middle groups after 2003–4. This implies that the related promotion programs did not effectively reach the people in this community. Moreover, lower income status might be an additional risk factor for poor oral hygiene, thereby further increasing the risk of oral cancer among the disadvantaged females. Because early detection and effective treatment has been shown to prevent at least one third of all cancers worldwide, including oral cancer [27], we recommend continued efforts be made on prevention and monitoring of incidence rates.

### Possible factors of health inequality

One previous literature reported low income as an independent risk factor for cancer epidemiology [16]. Our study corroborates the finding by showing that increased lifetime risk of oral cancer appears to be associated with poorer groups, even with universal healthcare coverage. The International Agency for Research on Cancer has found that cigarette smoking, betel quid chewing, and alcohol consumption clearly relate to the development of oral cancer [28]. In particular, chewing betel quid is closely associated with oral cancer in Taiwan, India, Thailand, and Pakistan [29]. This habit is also more prevalent among low income men and women in Taiwan, which reveals a possible causal association between betel quid chewing and oral cancer among the economically disadvantaged population. According to the serial data collected from the national health surveys of Taiwan, betel quid chewing rates of people living in high-, middle-, and low-income areas during the last decade were as follows[http://nhis.nhri.org.tw/2001nhis.htm;http://www.hpa.gov.tw/BHPNet/Web/healthtopic/TopicArticle.aspx?No=200712270003&parentid=200712270001]: males—9.23%, 11.45%, and 18.84% (2001); 13.24%, 16.22%, and 21.51 (2002); 9.95%, 13.42%, and 20.61% (2005); females—0.37%, 0.34%, and 4.08% (2001); 0.70%, 0.36%, and 2.71% (2002); 0.34%, 0.36%, and 2.29% (2005). They seem to be in sync with those depicted in Fig 4. This behavior is consistent with findings reported in China and Malaysia [30]. We suspect that the related public health education efforts in Taiwan may not have effectively reached more economically disadvantaged groups, and thus recommend more work be undertaken to reduce betel quid chewing rates in this community [31].

## Conclusions

Based on individual income and area of residence, our research results indicate that the implementation of the NHI has reduced related health inequalities by only a limited extent. Although universal coverage may improve the accessibility and quality of care, as indicated by the reduction in the difference in EYLL among different income groups (Table 2 and Fig 3), it has not reduced the occurrence of new cases of oral cancer in the low-income group, as found in this study (Fig 4). Life-long risk of oral squamous cell carcinoma (OSCC) depends on awareness and change of health behavior. These results highlight the need for improving the health literacy and behavior among poorer populations in order to reduce the incidence of oral cancer. Thus, the Taiwan government should invest more on prevention services to tackle this problem of health inequality.

## Supporting information

S1 Fig Data application procedures. (TIF)

## References

[1] Morton A. Aversion to health inequalities in healthcare prioritization: A multicriteria optimization perspective. J Health Econ 2014; 36: 164–173. 10.1016/j.jhealeco.2014.04.005 24831800

[2] Wagstaff A, van Doorslaer E. Measuring and testing for inequity in the delivery of health care. J Hum Resour 2000; 35: 716–733.

[3] Currie J. Socio-economic status and child health: does public health insurance narrow the gap? The Scand J Econ 1995; 97: 603–620.

[4] Decker S, Remler D. How much might universal health insurance reduce socioeconomic disparities in health? A comparison of the US and Canada. Appl Health Econ Health Policy 2004; 3: 205–216. 15901195 10.2165/00148365-200403040-00004

[5] Curtis L, Dooley M, Lipman E, Feeny D. The role of permanent income and family structure in the determination of child health in Canada. Health Econ 2001; 10, 287–302. 10.1002/hec.591 11400252

[6] Chang H, Chou Y. Amending health disparities in Taiwan’s indigenous population. Harvard Health Policy Rev 2002; 3: 40–46.

[7] Knesebeck O, Lüschen G, Cockerham W, Siegrist J. Socioeconomic status and health among the aged in the United States and Germany: a comparative cross-sectional study. Soc Sci Med 2003; 60: 1643–1652. 10.1016/s0277-9536(03)00020-0 12948573

[8] Wen C, Tsai S, Chung W. A 10-year experience with universal health insurance in Taiwan: measuring changes in health and health disparity. Ann Intern Med 2008; 148: 258–267. 18283203 10.7326/0003-4819-148-4-200802190-00004

[9] Hwang J, Wang JD. Monte Carlo estimation of extrapolation of quality-adjusted survival for follow-up studies. Stat Med 1999; 18: 1627–1640. 10407234 10.1002/(sici)1097-0258(19990715)18:13<1627::aid-sim159>3.0.co;2-d

[10] Fang C, Chang Y, Hsu H, Tu S, Chen K, Lin C, et al Life expectancy of patients with newly-diagnosed HIV infection in the era of highly active antiretroviral. QJM 2007; 100: 97–105. 10.1093/qjmed/hcl141 17277317

[11] Andersson TML, Dickman PW, Eloranta S, Lambe M, Lambert PC. Estimating the loss in expectation of life due to cancer using flexible parametric survival models. Stat Med 2013; 32: 5286–5300. 10.1002/sim.5943 24038155

[12] Chu P, Wang J, Hwang J, Chang Y. Estimation of life expectancy and the expected years of life lost in patients with major cancers: extrapolation of survival curves under high-censored rates. Value Health 2008; 11: 1102–1109. 10.1111/j.1524-4733.2008.00350.x 18489497

[13] Jackson C, Stevens J, Ren S, Latimer N, Bojke L, Manca A, et al Extrapolating survival from randomized trials using external data: A review of methods. Med Decis Making 2017; 37: 377–390. 10.1177/0272989X16639900 27005519 PMC5424081

[14] Cheng S, Chiang T. Disparity of medical care utilization among different health insurance schemes in Taiwan. Soc Sci Med 1998; 47: 613–620. 9690844 10.1016/s0277-9536(98)00103-8

[15] Hung MC, Lai WW, Chen HHW, Su WC, Wang JD. Comparison of expected health impacts for major cancers: integration of incidence rate and loss of quality-adjusted life expectancy. Cancer Epidemiol 2015; 39:126–132 10.1016/j.canep.2014.12.004 25553846

[16] Donnelly D, Gavin A. Socio-economic inequalities in cancer incidence-The choice of deprivation measure matters. Cancer Epidem 2011; 35: 55–61. 10.1016/j.canep.2011.06.002 21840786

[17] Chen Y, Huang Y, Lin L, Lin C. Primary oral squamous cell carcinoma: an analysis of 703 cases in southern Taiwan. Oral Oncol 1999; 35: 193–179. 10.1016/s1368-8375(98)00101-8 10435152

[18] Ko Y, Huang Y, Lee C, Chen M, Lin L, Tsai C. Betel quid chewing, cigarette smoking and alcohol consumption related to oral cancer in Taiwan. J Oral Path Med 2006; 24: 450–453. 10.1111/j.1600-0714.1995.tb01132.x 8600280

[19] Wong Y, Tsai W, Lin J, Poon C, Chao S, Hsiao Y, et al Socio-demographic factors in the prognosis of oral cancer patients. Oral Oncol 2006; 42: 839–906. 10.1016/j.oraloncology.2005.12.007 16730220

[20] The World Health Organization. International classification of diseases for oncology (ICD-O)– 3rd edition, 1st revision, 2013.

[21] Directorate-General of Budget, Accounting and Statistics, Executive Yuan, Taiwan. Survey of Family Income and Expenditure, 2016, retrieved on March 30, 2017 from http://win.dgbas.gov.tw/files/214.asp.

[22] Department of Health and Welfare, Executive Yuan, Taiwan. Performance of Prevention on All Cancers: 5-year Program Report. Taipei, Taiwan, 2008.

[23] Burnet NG, Jefferies SJ, Benson RJ, Hunt DP, Treasure FP. Years of life lost (YLL) from cancer is an important measure of population burden—and should be considered when allocating research funds. Brit J Cancer 2005; 92: 241–245. 10.1038/sj.bjc.6602321 15655548 PMC2361853

[24] Yang SC, Lai WW, Lin CC, Su WC, Ku LJ, Hwang JS, et al Cost-effectiveness of implementing computed tomography screening for lung cancer in Taiwan. Lung Cancer 2017; 108:183–191. 10.1016/j.lungcan.2017.04.001 28625633

[25] Davis W. Cancer registration and its techniques. Lyon: International Agency for Research on Cancer, 1978.

[26] Rachet B, Ellis L, Maringe C, Chu T, Nur U, Quaresma M, et al Socioeconomic inequalities in cancer survival in England after the NHS cancer plan. Brit J Cancer 2010; 103: 446–453. 10.1038/sj.bjc.6605752 20588275 PMC2939774

[27] Petersen P. Oral cancer prevention and control-The approach of the World Health Organization. Oral Oncol 2009; 45: 454–460. 10.1016/j.oraloncology.2008.05.023 18804412

[28] International Agency for Research on Cancer. Betel-quid and areca-nut chewing and some areca-nut-derived Nitrosamines In: IARC Monographs on the Evaluation of Carcinogenic Risks to Humans. France: World Health Organization International Agency for Research on Cancer 85, 2003. PMC4781453 15635762

[29] Akhtar S. Areca nut chewing and esophageal squamous-cell carcinoma risk in Asians: A meta-analysis of case-control studies. Cancer Cause Control 2013; 24: 257–265. 10.1007/s10552-012-0113-9 23224324

[30] Ghani W, Razak I, Yang Y, Talib N, Ikeda N, Axell T, et al Factors affecting commencement and cessation of betel quid chewing behavior in Malaysian adults. BMC Public Health 2011; 11: 82–89 10.1186/1471-2458-11-82 21294919 PMC3039591

[31] Smith KE, Stewart E, Donnelly P, Mckendrick B. Influencing policy with research-public health advocacy and health inequalities In Health Inequalities: Critical Perspectives eds. By Smith KE, Hill S, Bambra C, Oxford University Press: 265–278, 2016.

