# Supplementary figures and images for: Universal health insurance, health inequality and oral cancer in Taiwan
Source: PLoS One. 2018 Oct 18;13(10):e0205731. doi: 10.1371/journal.pone.0205731 (PMC6193672; doi:10.1371/journal.pone.0205731)

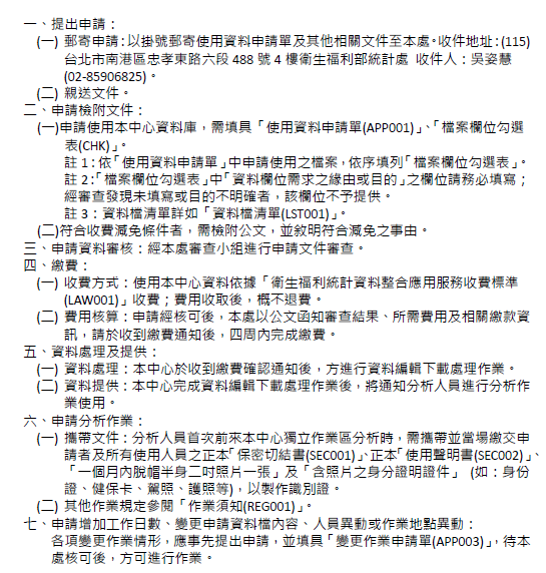

Supplement: S1 Fig — (TIF) [file pone.0205731.s001.tif]
